# 2025 Targeting immunosuppressive myeloid cells to enhance cancer immunotherapy

**DOI:** 10.1017/cts.2018.127

**Published:** 2018-11-21

**Authors:** Xin Lu

**Affiliations:** Indiana University School of Medicine

## Abstract

OBJECTIVES/SPECIFIC AIMS: Prostate cancer (PCa) is the most common noncutaneous malignancy in men in the United States. A significant fraction of advanced PCa treated with androgen deprivation therapy experience relentless progression to lethal metastatic castration-resistant prostate cancer (mCRPC). The PCa tumor microenvironment is comprised of a complex mixture of epithelial and stroma cell types engaged in multifaceted heterotypic interactions functioning to maintain tumor growth and immune evasion. We recently uncovered the important role played by myeloid-derived suppressor cells (MDSCs) to mediate tumor immune evasion in aggressive PCa (Wang, Lu *et al*., Cancer Discovery, 2016). Immune checkpoint blockade (ICB) has elicited durable therapeutic responses across a number of cancer types. However, the impact of ICB on mCRPC has been disappointing, which may signal the need to combine mechanistically-distinct ICB agents and/or override immunosuppression in the tumor microenvironment. Our objective is to determine if robust immunotherapy responses in mCRPC may be elicited by the combined actions of ICB agents together with targeted agents that neutralize MDSCs yet preserve T cell function. METHODS/STUDY POPULATION: We created a novel embryonic stem cell-based chimeric mouse model of mCRPC engineered with signature mutations to study the response to single and combination immunotherapy. The efficacy studies were followed with detailed mechanistic investigation and clinical validation. RESULTS/ANTICIPATED RESULTS: Consonant with early stage clinical trials experience, anti-CTLA4 or anti-PD1 monotherapy failed to impact disease progression. Similarly, modest anti-tumor activity was observed with combination ICB as well as monotherapy with targeted agents including Cabozantinib (tyrosine kinase inhibitor), Dactolisib (PI3K/mTOR inhibitor), and Dasatinib (tyrosine kinase inhibitor). In contrast, mCRPC primary and metastatic disease showed robust responses to dual ICB treatment together with either Cabozantinib or Dactolisib, but not with Dasatinib which impaired T cell infiltration in the tumor. Detailed intratumoral immune profiling with mass cytometry (CyTOF) showed that combined ICB and Cabozantinib or Dactolisib was associated with significant depletion of MDSCs. Cabozantinib and Dactolisib blocked the PI3K signaling activity in MDSCs and reduced their immunosupppresive activity. Mechanistically, the combination efficacy was due to the upregulation of IL-1RA and suppression of MDSC-promoting cytokines secreted by mCRPC cells. DISCUSSION/SIGNIFICANCE OF IMPACT: We demonstrated that an antibody cocktail targeting CTLA4 and PD1 was insufficient to generate effective anti-tumor response, but combination of ICB with targeted therapy that inactivates PI3K signaling displayed superior synergistic efficacy through impairing MDSCs in the tumor microenvironment. These observations illuminate a clinical path hypothesis for combining ICB with MDSC-targeted therapies in the treatment of mCRPC. Importantly, conclusions from the study on PCa may have implications in combination immunotherapy for aggressive breast cancer which is also largely resistant to immune checkpoint blockade and replete with immunosuppressive myeloid cells.

